# The Anyang Esophageal Cancer Cohort Study: Study Design, Implementation of Fieldwork, and Use of Computer-Aided Survey System

**DOI:** 10.1371/journal.pone.0031602

**Published:** 2012-02-06

**Authors:** Fangfang Liu, Fangcen Guo, Yue Zhou, Zhonghu He, Xiuyun Tian, Chuanhai Guo, Tao Ning, Yaqi Pan, Hong Cai, Yang Ke

**Affiliations:** 1 Key Laboratory of Carcinogenesis and Translational Research, Ministry of Education, Peking University School of Oncology, Beijing Cancer Hospital and Institute, Beijing, People's Republic of China; 2 National Research Institute for Family Planning, Beijing, People's Republic of China; Institut Gustave Roussy, France

## Abstract

**Background:**

Human papillomavirus (HPV) has been observed repeatedly in esophageal squamous cell carcinoma (ESCC) tissues. However, the causal relationship between HPV infection and the onset of ESCC remains unknown. A large cohort study focusing on this topic is being carried out in rural Anyang, China.

**Methodology/Principal Findings:**

The Anyang Esophageal Cancer Cohort Study (AECCS) is a population-based prospective endoscopic cohort study designed to investigate the association of HPV infection and ESCC. This paper provides information regarding the design and implementation of this study. In particular we describe the recruitment strategies and quality control procedures which have been put into place, and the custom designed computer-aided survey system (CASS) used for this project. This system integrates barcode technology and unique identification numbers, and has been developed to facilitate real-time data management throughout the workflow using a wireless local area network. A total of 8,112 (75.3%) of invited subjects participated in the baseline endoscopic examination; of those invited two years later to take part in the first cycle of follow-up, 91.9% have complied.

**Conclusions/Significance:**

The AECCS study has high potential for evaluating the causal relationship between HPV infection and the occurrence of ESCC. The experience in setting up the AECCS may be beneficial for others planning to initiate similar epidemiological studies in developing countries.

## Introduction

Esophageal cancer is a common and often fatal malignancy, and it shows a striking geographic variation in incidence [1], [2]. One of the highest incidence regions for this neoplasm is in the Anyang region of Northern China, and the major etiologic factors for development of esophageal cancer in this region remain to be determined [3], [4], [5], [6], [7].

The association between human papillomavirus (HPV) infection and esophageal squamous cell carcinoma (ESCC) has been under investigation since the early 1980s, and results have been inconsistent [8], [9], [10], [11], [12], [13], [14]. We have documented the presence of HPV DNA in many esophageal cancer specimens collected in Anyang [15]. Moreover, a case-control study in this population observed that the presence of HPV DNA in cancer specimens and the presence of HPV-16 E7 antibody in sera were associated with an increased risk of ESCC (Unpublished manuscript). In order to further investigate the causal relationship between HPV infection and the incidence of ESCC, the Anyang Esophageal Cancer Cohort Study (AECCS) was launched in 2006. The AECCS seeks to: 1) explore the natural history of HPV infection in esophageal epithelium; 2) investigate modes of transmission of HPV infection to the esophagus; and 3) evaluate the association between HPV infection and the occurrence of ESCC.

The design and implementation of this prospective cohort study has posed numerous challenges. First, a large sample size and long duration of follow-up are required due to the low incidence of ESCC [16]. Second, in order to evaluate the possibility of spread of HPV infection from one part of the body to another, examination of multiple sites on each individual for HPV in addition to the esophagus is essential, and this complicates study procedures and data management. Third, the study is being carried out in a region of limited resources where some modern technologies (such as stable Internet connections) are not available [17], [18]. The relatively low educational level of participants has also posed a challenge [19]. In this article we seek to describe the design and implementation of the AECCS.

## Methods

### Study design

The primary exposure to be investigated in the AECCS is esophageal HPV infection, determined by evaluating the HPV DNA status of endoscopic biopsy specimens using polymerase chain reaction (PCR) followed by sequencing and the HPV antibody status in sera using enzyme-linked immunosorbent assay (ELISA) [15]. The primary endpoint is the presence of ESCC/precancerous lesions (identified by endoscopic examination and subsequent pathological diagnosis of biopsy tissues [20]) or clinically diagnosed ESCC. The AECCS is being conducted in Anyang, the northernmost area of Henan Province, China. Anyang consists of five rural counties, including Anyang, Hua, Lin, Neihuang, and Tangyin, which have a total of 4.37 million people. The mortality rate for ESCC in this area is about 50 per 100,000 person-years [21]. Anyang is an agricultural region of low-income with a per capita gross domestic product (GDP) of $1,760 (USD). Like other rural areas of China, more than 94% of rural populations in Anyang are covered by New Rural Cooperative Medical Scheme (NRCMS, a government-run voluntary insurance) and the inpatient reimbursement proportion is about 50% on average [22]. To represent the spectrum of villages in Anyang according to location, population size, administrative capabilities, etc., five villages (Hengcun, Malan, Xiaopu, Xitaoyuan and Zhuzhao) from Hua County, two (Chengbei and Shencun) from Lin County (also called Linxian, or Linzhou), one (Shangzhuang) from Anyang County and one (Tunzhuang) from Tangyin County were selected for inclusion in the study. Eligibility criteria for participation were as follows: 1) permanent residency in one of the selected villages; 2) age between 25 and 65 years; 3) no self-reported history of cancer, cardiovascular disease, mental disorder, or other contraindications for endoscopy; 4) no self-reported history of infection with hepatitis B virus (HBV), hepatitis C virus (HCV), or human immunodeficiency virus (HIV) (and no evidence of these infections based on serum screening); and 5) willingness to participate in AECCS and complete all parts of the examination. Over 8,000 residents were eligible to be recruited into the cohort, and follow-up is planned for a 10-year period at 2-year intervals. All medical procedures carried out in the AECCS are free of charge, no financial or other incentive is given to participants, except a light meal after completion of examinations. The ESCC patients identified in this cohort are given RMB 1,000 (1,000 Renminbi≈$150 USD) to reduce the financial burden of cancer treatment. This study was conducted according to the principles expressed in the Declaration of Helsinki. Every attempt to protect participants' privacy and confidentiality has been made, including labeling specimens with a unique number without exposing any personal information and publishing anonymously. Research protocols and related materials were submitted to and approved by an independent ethics committee, the Institutional Review Board of the School of Oncology, Peking University, China (Approval number: 2006020). All participants provided written informed consent.

### Organization of fieldwork

**Figure 1:**
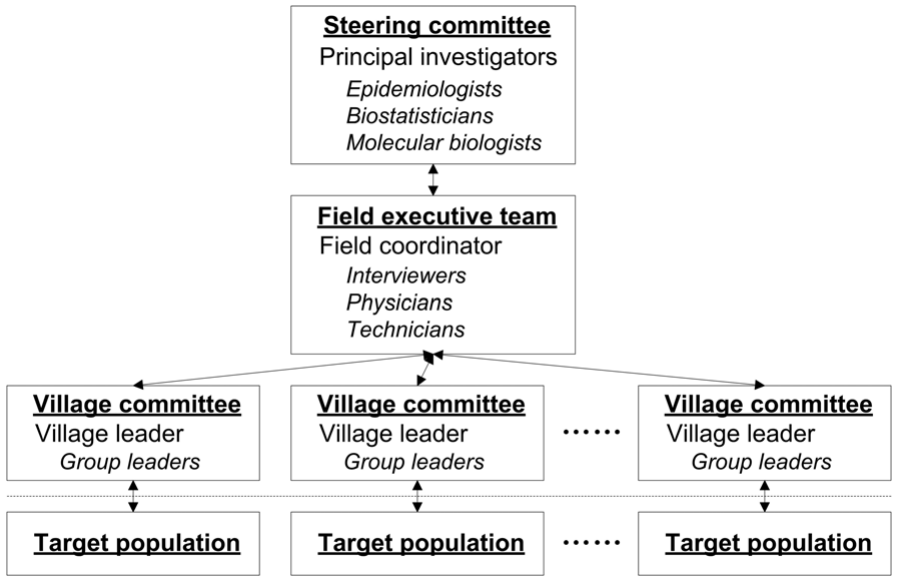
Three-level infrastructure of the AECCS. This figure illustrates how three tiers of responsibility are integrated in the fieldwork infrastructure for optimal interaction with the target population in the Anyang Esophageal Cancer Cohort Study, Anyang, China, 2006-present. There are a total of nine village committees in the third tier of the infrastructure.

The AECCS fieldwork infrastructure includes three tiers of responsibility (Figure 1). The first tier is the steering committee which consists of the Principal Investigators and scientific experts who are responsible for study design and decisions regarding study implementation. The second tier is the fieldwork executive team, which consists mainly of local dialect-speaking staff who have medical backgrounds. This tier, led by the field coordinator, carries out the routine survey procedures in the AECCS study center and oversees specific elements of the fieldwork through contact with village committees. The study center resides in Hua County Hospital, the largest general hospital in the study region, and this hospital is located an average of 25 miles from the villages selected for study. A motor vehicle is employed for transportation of participants back and forth from the study center. The study center is fully supported by Hua County Hospital and is allowed access to its clinical laboratory, emergency room and supply division. Nine village committees represent the third tier of the infrastructure and play a critical role in participant recruitment. Each village committee consists of the administrative head of the village together with subordinate group leaders of the neighborhood. The size of these subordinate groups ranges from 50 to 200 residents, and group leaders are familiar with resident information and are able to contact individuals as need be in the target population. Village committees are also responsible for monitoring the occurrence of clinically diagnosed cancers, whereabouts, and vital status of cohort members.

### Participant recruitment

The original recruitment process was led by the field coordinator and carried out by village committees. The recruitment event was first announced by village leaders and bulletins were displayed in all villages. This was followed by house-to-house recruitment carried out by group leaders based on a list of potential participants obtained from village security offices. During home visits, group leaders described the study in a more detailed manner and answered questions. Residents without any self-reported history of conditions which would render them ineligible were invited to attend a hospital appointment in a non-coercive way. If villagers were not at home at the first visit, 2 additional attempts were made. Permanent residents who were temporarily away (e.g. working elsewhere) were recruited by telephone and flexible schedules were arranged. Village committees met with the field coordinator on a bi-weekly basis to provide information on the progress of recruitment, to discuss specific problems, and to schedule visits.

### Baseline investigation

#### Registration and blood screening

Demographic information for potential study participants including name, gender and date of birth was obtained from village security offices and preloaded into the study database. When participants presented to the study center, a personal identification card issued by the Chinese government was required. After confirmation of identity and contact information, a digital photograph was taken, and a unique study identification (ID) number and barcode were automatically generated by the database. Investigators told the potential participants about the discomforts and risks of all involved examinations, and ensured them informed that participation is voluntary and non participation or withdrawal will not result in any penalty or loss of benefits to which they are otherwise entitled. An informed consent statement was then signed by each invitee who decided to take part in the study. Each participant gave two blood samples. One sample was used for screening for HBV, HCV, and HIV infection, and individuals with evidence of any of these infections were excluded from the cohort. The other blood sample (5 ml) was centrifuged, and serum and blood cells were temporarily stored at −20°C and later transferred to Beijing Cancer Hospital and Institute, and frozen at −80°C.

#### Interview

A one-on-one interview was carried out by a trained interviewer. The electronic questionnaire, which is linked with the study database, includes more than 50 items regarding demographic characteristics, medical history, family history of cancer, personal behavior (e.g. smoking and drinking), dietary habits, hygienic habits, sexual behavior, and for women, menstrual/reproductive history.

#### Collection of exfoliated cells

Exfoliated cells of oral cavity, palmar skin, and genitalia were collected (Collection of palmar exfoliated cells and male genital exfoliated cells was limited to 6 villages during baseline evaluation). Oral exfoliated cells were collected from inner cheeks, inner lower and upper lip, palate, and surface of the tongue by swabbing five times after rinsing the mouth. Palmar exfoliated cells were collected from the entire surface of the palm by scraping with steady pressure. Exfoliated cells were also obtained from the genitalia. For women, a gynecologic examination was performed before cell collection, and any abnormalities were recorded. Cervical secretions were cleared, and then the exfoliated cells were collected by insertion of swab into the cervix and rotation of 360° five times. Cells were immediately smeared onto a slide and fixed for cytological examination, and the swab was then processed for cells for further analysis. For men, the upper 1/3 of the scrotum and entire surface of the penile shaft, coronal sulcus and glans penis were swabbed five times.

Exfoliated cells collected on swabs were rinsed into 0.9% saline solution and centrifuged. Cell pellets were first frozen at −20°C and later at −80°C.

#### Endoscopic biopsy

The esophagus and stomach were inspected via endoscopy and all abnormalities were biopsied. If no lesions were found, a biopsy was taken at the mid-esophagus for pathologic examination. In addition, biopsies from the 6 and 12 o'clock positions 25 cm distal to the incisors were taken for HPV testing. Using the endoscopic imaging system, an electronic endoscopy report was completed and stored in the database. A copy of the report was given to the participant.

### Follow-up

The participant roster is updated every 2 years, with addition of new qualified residents (e.g. new 25-year-old residents) and removal of participants who have reached study end-points in the previous cross-section. All subjects on the roster are contacted for follow-up examinations, which are identical to those performed for the baseline study except that the follow-up questionnaire mainly focuses on changes in exposures of ESCC or HPV relevant factors in the preceding 2-year interval. Information on incident clinically diagnosed cancers, deaths, and migration occurred during the interval since the previous cross-section is also obtained through follow-up home visits. Death certificates are then requested from local medical service units to verify the date and cause of death. For cohort members who are newly diagnosed with ESCC, relevant medical records, histology slides, and tumor tissue blocks are collected from the diagnostic hospital for confirmation of cancer diagnosis and future studies.

### Computer-aided survey system

**Figure 2:**
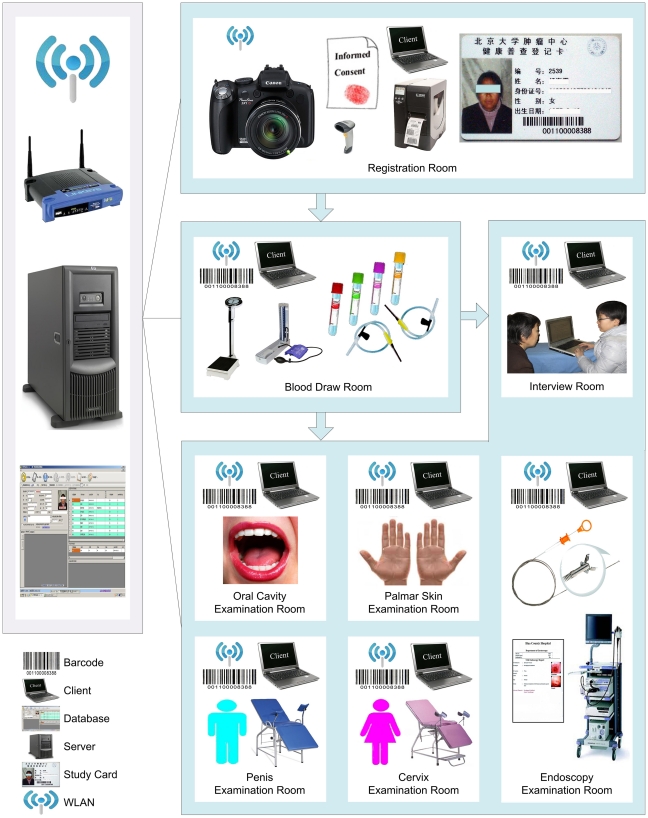
Computer-aided survey system (CASS) with client/server mode using a WLAN. The hosting system for the database resides on the server. The staff in each examination room can synchronously enter data in the database by logging into client application software installed on client computers using a wireless local area network (WLAN). After registration, blood is drawn. Eligible participants with no communicable blood diseases then continue on and participate in an interview and examination of the oral cavity, palmar skin, cervix/penis and esophagus.

In order to facilitate data collection, data management, and quality control, particular effort was taken in developing a custom designed computer-aided survey system (CASS). To support the synchronization and sharing of data across multiple examination rooms without the benefit of Internet access, information technologies including use of barcodes, ID numbers, and wireless local area network (WLAN) were integrated into CASS (Figure 2). Data can thus be entered and retrieved in the Structured Query Language (SQL) Server 2005 database via logging into client computers. Thus, the CASS server/client mode allows real-time data entry and management, and permits all procedures to run simultaneously.

At the same time, this information system allows for dynamic monitoring of study progress. For example, up-to-date survey status information can be accessed by the study staff to identify a participant whose data are incomplete so that information can be amended even before the individual leaves the study center. This system also makes it possible to rapidly produce statistical reports to order in order to obtain an overview of the study while it is in progress. On the other hand, to ensure the completeness, accuracy and consistency of data, check functions and skip patterns were programmed into the database wherever possible. For example, completion of all the questions is required before questionnaires may be submitted to the database.

### Quality control

To minimize variation in implementation of procedures by various staff members over time, standardized operation protocols (SOPs) have been developed, and personnel are trained and certified according to these SOPs. In particular, since HPV cross-contamination can adversely influence the accuracy of results, SOPs detailing anti-contamination measures have been established. In addition, procedures evaluating the effectiveness of anti-contamination have been included. Mock samples, such as mouse liver free of HPV, are processed together with biopsies and cells, and swabs of the working environment are routinely collected [23]. Periodically, all these mouse liver and swab samples are tested for human beta-globin and HPV DNA negativity. Random samples of tape-recorded interviews are also routinely reviewed and evaluated for protocol adherence.

## Results

**Figure 3:**
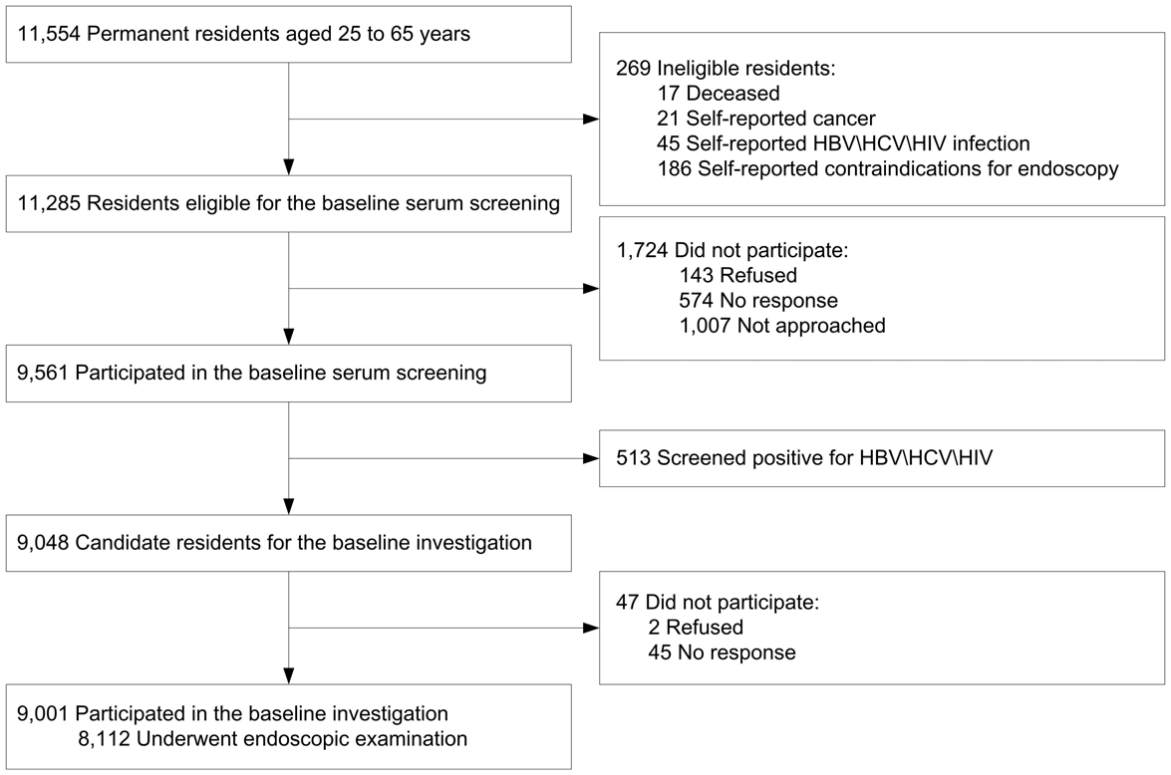
Flow diagram of participants. This figure illustrates the flow diagram of participants through enrollment, serum screening and endoscopic examination in the baseline investigation of Anyang Esophageal Cancer Cohort Study, Anyang, China, 2006–2009.

In the baseline phase of the AECCS (2006 to 2009), of 11,554 permanent residents from selected villages of ages ranging from 25 to 65, 269 were excluded because of death and self-reported ineligibility (Figure 3). Of the remaining 11,285 candidate residents, 9,561 (84.7%) had blood drawn. Of these individuals, 513 (5.4%) were excluded from the cohort, including 414 who were positive for HBV, 96 for HCV and 3 for HIV. Thus, 10,772 (11,285 minus 513) residents were included as eligible. Of these potential participants, 8,638 (80.2%) completed a baseline interview and 8,112 participated in an endoscopic examination, yielding a final response proportion of 75.3% (8,112/10,772) (Table 1). The response proportions for examination of oral cavity, palmar skin, cervix and penis were 75.3%, 82.6%, 77.2%, and 84.6%.

**Table 1:** Number of participants and response proportions in the baseline investigation, Anyang Esophageal Cancer Cohort Study (2006–2009).

Examination items	Eligible subjects	Participants	Response proportions
	No.	No.	Percent
Interview	10,772	8,638	80.2
Endoscopic examination	10,772	8,112	75.3
Examination of oral cavity	10,772	8,109	75.3
Examination of palmar skin†	7,210	5,958	82.6
Examination of cervix‡	5,401	4,171	77.2
Examination of penis†	3,571	3,021	84.6

† Examination of palmar skin and penis was limited to 6 villages (Hengcun, Malan, Tunzhuang, Xiaopu, Xitaoyuan, and Zhuzhao) in the baseline investigation.

‡ Fifty women with previous hysterectomies did not have a cervical examination.

The mean age of participants who underwent baseline endoscopy was 44.3 years (Standard deviation 10.5 years) and the male to female sex ratio was 1.00 to 1.11 (Table 2). More than 40% of these participants had not attended high school or higher levels of education and about 60% were currently farming for their livelihood. Approximately 7.6% reported having a family history of esophageal cancer. In the baseline study, 20 ESCCs, 2 esophageal adenocarcinomas, 4 cardiac adenocarcinomas, 1 gastric adenocarcinomas, 1 duodenal adenocarcinoma and 14 cervical carcinomas were identified.

**Table 2:** Selected characteristics of endoscopy participants in the baseline investigation, Anyang Esophageal Cancer Cohort Study (2006–2009).

Variables	No. of subjects	Percent
	(N = 8,112)	
Village		
Chengbei	803	9.9
Hengcun	918	11.3
Malan	1,378	17.0
Shangzhuang	681	8.4
Shencun	654	8.1
Tunzhuang	1,389	17.1
Xiaopu	702	8.7
Xitaoyuan	409	5.0
Zhuzhao	1,178	14.5
Age (years)		
25–35	1,843	22.7
36–45	2,889	35.6
46–55	1,916	23.6
56–65	1,464	18.0
Mean age (Standard deviation)	44.3 (10.5)	
Gender		
Female	4,268	52.6
Male	3,844	47.4
Education		
Illiterate	959	11.8
Primary school	2,540	31.3
Junior high school	3,496	43.1
Senior high school or above	668	8.2
Unknown	449	5.5
Type of employment		
Farming	5,062	62.4
Non-farming	2,601	32.1
Unknown	449	5.5
Family history of esophageal cancer		
No	7,201	88.8
Yes	613	7.6
Unknown	298	3.7

The first follow-up for the AECCS began in 2009, and to date follow-up has been completed for 3 villages. Of the 2,899 eligible residents from these 3 villages, 2,664 (91.9%) have undergone a follow-up endoscopic examination.

## Discussion

This paper describes the design and implementation of the AECCS, which is one of the largest prospective esophagogastroduodenoscopic cohort studies undertaken to date. In view of the fact the cohort is large, the frequency of study follow up is relatively high, and the length of follow-up is long, this study has high potential for evaluating the relationship between esophageal HPV infection and the occurrence of ESCC. To maximize the validity of this study, carefully-designed recruitment strategies and a tailored computer-aided survey system have been employed. Participation and retention have been high, as reflected by the fact that 8,112 (75.3%) participants underwent baseline endoscopic examination and 91.9% of invited subjects have, to date, participated in the first cycle of follow-up. In view of the initial success of this study, the methodology we have employed for implementing AECCS may provide benefit for others who plan to launch similar large-scale epidemiological studies in low-resource regions.

Based on data from our case-control study mentioned above, the proportion of individuals positive for HPV DNA in the normal esophagus in this region is expected to be about 5%. Due to the repeated and transient nature of infection characteristic of HPV, we estimate that 15% of subjects will have been characterized as HPV-exposed after a 10-year follow-up. In addition, since ESCC has a very poor prognosis, the incidence rate is approximately equal to the mortality rate which is about 50 per 100,000 person-years [21], [24]. Moreover, we estimate that there will be a 10% loss of participants at every cross-sectional examination, so that some malignant lesions that do not progress to clinically-evident cancer will be missed. Therefore, using a two-sided log-rank test on the significance level of 0.05 and an enrollment of 8,112 subjects in the baseline investigation, the AECCS would have 80% power to detect risk ratios greater than 3.0 (PASS 2008 software) (Figure S1). The odds ratio of HPV exposure obtained in our case-control study was approximately 6.0. Thus, the current sample size of this cohort can be considered adequate for evaluating the relationship of esophageal HPV infection and the onset of ESCC (Table S1).

In the AECCS, population-based endoscopic examination is carried out biennially in participants across a wide age range (25–65 years). The inclusion of younger participants is anticipated to yield valuable data regarding HPV infection and onset of esophageal lesions. Ideally, we would have included even younger people in order to capture the time of onset of sexual activity for measurement of HPV infection [25]. However, requesting large number of individuals under age 25 to undergo endoscopy is not feasible. Due to the transient nature of HPV infection, follow-up with repeated examination to ascertain HPV infection status of an individual will reduce misclassification of exposure [26], [27], [28]. At the same time, as the study size is large and the age range is wide, related studies can be incorporated in a cost-effective way. Examinations of the oral cavity, palmar skin, and genitals are carried out simultaneously, which enables investigation of outcomes in other organ systems such as the female genital tract and may allow us to study modes of transmission of HPV infection to the esophagus.

As a rule of thumb, a high response proportion is an indicator of good survey practice [29]. Compared to some studies with lower participant burden, AECCS requires relatively substantial time commitments and involves invasive procedures [30]. Therefore, more efforts are demanded to achieve a satisfied participation proportion. The attained high level of response to AECCS recruitment and follow-up can be attributed to several factors. First, there has been full support from local health providers and the local government. For example, establishment of the study center in the local hospital has provided participants a safe and comfortable environment for required examinations which has improved recruitment. Second, well organized fieldwork has been vital to the success of recruitment. The fieldwork coordinator has excellent skills in working with local residents and influential members of the village committees, and has served as a strong bridge between investigators and the target population. Thus, culturally appropriate, non-coercive recruitment strategies have been developed to motivate participation and to avoid misunderstanding. In addition, regular meetings to discuss progress and challenges have helped sustain and promote recruitment. Last but not least, performance quality and the friendly attitude of study personnel have also been crucial in this low medical resource region. Professional physicians and other staff have been continuously available to explain examination results and to answer other medical questions from participants. We believe that participants thus have had generally positive experiences and frequently shared their trust in our work with other invitees. Altogether, while our experience should not be regarded as an exact recruitment recipe, it is clear that crafting and using study-specific strategies to reconcile the unique cultural barriers and concerns will be paramount to success in recruitment and retention [31].

The successful use of CASS in this cohort also deserves mention. First, use of CASS has allowed high-quality data to be obtained, stored and retrieved in an efficient, inexpensive manner. The incorporation of ID numbers, barcodes and photographs throughout the workflow has ensured accurate subject identification, which may be a serious challenge in resource-limited settings, and has significantly reduced potential manual input mistakes [19], [32]. Up-to-date retrieval of individual examination status has minimized accidental missing responses to examination items, and the built-in check functions have almost completely eliminated inaccuracies, inconsistencies, and incorrect data [33]. Second, CASS features real-time statistical evaluation of data enabling staff to efficiently and accurately produce various kinds of progress reports at any given time. Thus, labor intensive work, such us manually merging multiple databases to determine the progress of ongoing investigation, has been avoided. Use of this system has also ensured data security. Limitation of user's authority, use of WLAN to avoid public exposure of data, and routine database backup, as well as detailed emergency plans in event of accidental malfunction of CASS have all served to ensure data safety.

There are concerns which must be addressed regarding sustaining this cohort for ongoing study. Long-term retention of subjects will be one of the most challenging issues we may confront in the future. In the wake of economic growth in China, it is anticipated that there will be an increase in participants moving back and forth from their original residential place for reasons of employment. This will greatly increase the difficulty of retention of this cohort. It is clear that additional retention strategies and funding will be needed to maintain long-term follow-up.

## Supporting Information

Figure S1 **Setup of parameters to calculate sample size for AECCS (PASS 2008 software).** The follow-up of Anyang Esophageal Cancer Cohort Study (AECCS) is planned for a 10-year period at 2-year intervals. We estimate that there will be a 10% loss of participants at every cross-sectional examination (every 2 years), so proportion lost during 1 year is about 5.13% (See the following). The incidence rate of ESCC in unexposed group is 38.45 per 100,000 person-years (See the following). *Let P_lost1_ denote the proportion lost during 1 year and let P_lost2_ denote the proportion lost during 2 years (which is 10%), then (1−P_lost1_)∧2 = 1−P_lost2_ assuming that lost to follow-up is constant over time. Thus, P_lost1_ = 1−(1−10%)∧0.5 = 0.0513. Let P_0_ denote the incidence rate of ESCC in unexposed group, let P denote the incidence rate of the target population (which is 50 per 100,000 person-years), let RR denote risk ratio (which is 3.003) and let R denote sample size ratio of unexposed group (N_2_) to exposed group (N_1_) (which is equal to 85%/15% = 5.67), then P = (R×N_1_×P_0_+RR×N_1_×P_0_)/(R×N_1_+N_1_). Thus P_0_ = P×(1+R)/(R+RR) = 0.0005×(1+5.67)/(5.67+3.003) = 0.0003845.*
(TIF)

Table S1 **Statistical power calculation for Anyang Esophageal Cancer Cohort Study under different scenarios combining various values of sample size, proportion of exposed group and risk ratio.**
(DOC)
