# Supplementary figures and images for: The Anyang Esophageal Cancer Cohort Study: Study Design, Implementation of Fieldwork, and Use of Computer-Aided Survey System
Source: PLoS One. 2012 Feb 6;7(2):e31602. doi: 10.1371/journal.pone.0031602 (PMC3273470; doi:10.1371/journal.pone.0031602)

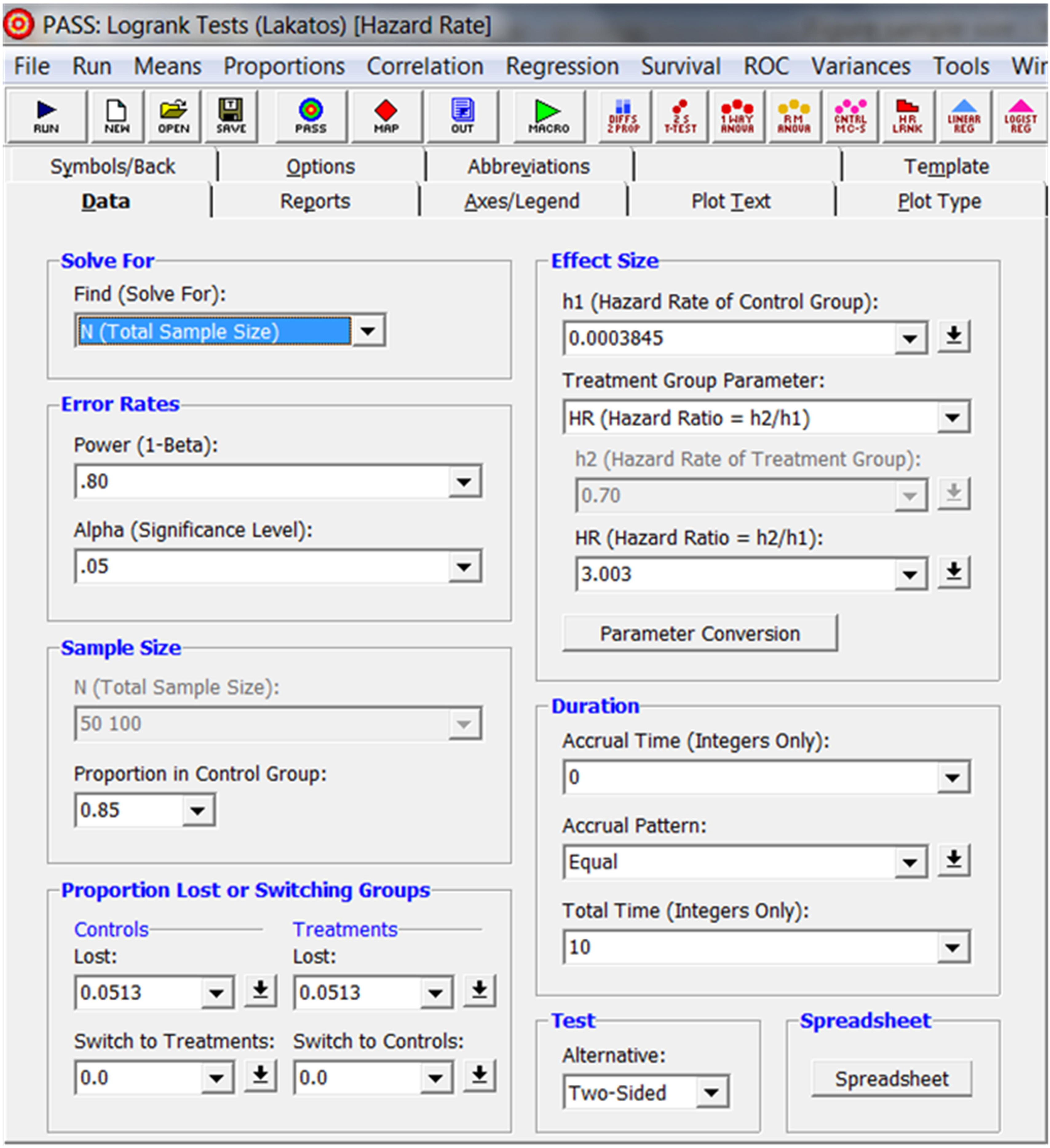

Supplement: Figure S1 — Setup of parameters to calculate sample size for AECCS (PASS 2008 software). The follow-up of Anyang Esophageal Cancer Cohort Study (AECCS) is planned for a 10-year period at 2-year intervals. We estimate that there will be a 10% loss of participants at every cross-sectional examination (every 2 years), so proportion lost during 1 year is about 5.13% (See the following). The incidence rate of ESCC in unexposed group is 38.45 per 100,000 person-years (See the following). Let Plost1 denote the proportion lost during 1 year and let Plost2 denote the proportion lost during 2 years (which is 10%), then (1−Plost1)∧2 = 1−Plost2 assuming that lost to follow-up is constant over time. Thus, Plost1 = 1−(1−10%)∧0.5 = 0.0513. Let P0 denote the incidence rate of ESCC in unexposed group, let P denote the incidence rate of the target population (which is 50 per 100,000 person-years), let RR denote risk ratio (which is 3.003) and let R denote sample size ratio of unexposed group (N2) to exposed group (N1) (which is equal to 85%/15% = 5.67), then P = (R×N1×P0+RR×N1×P0)/(R×N1+N1). Thus P0 = P×(1+R)/(R+RR) = 0.0005×(1+5.67)/(5.67+3.003) = 0.0003845. (TIF) [file pone.0031602.s001.tif]
