# Supplementary material for: The Anyang Esophageal Cancer Cohort Study: Study Design, Implementation of Fieldwork, and Use of Computer-Aided Survey System
Source: PLoS One. 2012 Feb 6;7(2):e31602. doi: 10.1371/journal.pone.0031602 (PMC3273470; doi:10.1371/journal.pone.0031602)
Supplement: Table S1 — Statistical power calculation for Anyang Esophageal Cancer Cohort Study under different scenarios combining various values of sample size, proportion of exposed group and risk ratio. (DOC) [file pone.0031602.s002.doc]

| Table S1. Statistical power calculation for Anyang Esophageal Cancer Cohort Study under different scenarios combining various values of sample size, proportion of exposed group and risk ratio† | | | | | |
| --- | --- | --- | --- | --- | --- |
| Sample size | Proportion of exposed group | Risk ratio | | | |
|  |  | 2.00 | 3.00 | 5.00 | 6.00 |
| 5,000 | 5% | 0.24 | 0.45 | 0.75 | 0.83 |
| 5,000 | 10% | 0.28 | 0.57 | 0.87 | 0.93 |
| 5,000 | 15% | 0.32 | 0.63 | 0.92 | 0.96 |
| 5,000 | 20% | 0.34 | 0.67 | 0.94 | 0.98 |
| 8,000 | 5% | 0.29 | 0.57 | 0.87 | 0.93 |
| 8,000 | 10% | 0.37 | 0.72 | 0.96 | 0.99 |
| 8,000 | 15% | 0.43 | 0.79 | 0.98 | 1.00 |
| 8,000 | 20% | 0.47 | 0.84 | 0.99 | 1.00 |
| 10,000 | 5% | 0.32 | 0.63 | 0.91 | 0.96 |
| 10,000 | 10% | 0.42 | 0.79 | 0.98 | 1.00 |
| 10,000 | 15% | 0.49 | 0.86 | 0.99 | 1.00 |
| 10,000 | 20% | 0.54 | 0.90 | 1.00 | 1.00 |

†Statistical power was calculated using a two-sided log-rank test on the significance level of 0.05 (PASS 2008 software). The incidence rate of the target population is 50 per 100,000 person-years and lost to follow-up (10-year period at 2-year intervals) is estimated to be 10% at every cross-sectional examination.
